# Efficacy of the silicone rubber uterine stent in the prevention of adhesion after hysteroscopic adhesiolysis: a prospective, randomized, controlled trial

**DOI:** 10.3389/fmed.2025.1629651

**Published:** 2025-11-26

**Authors:** Hanxiao Ding, Shikang Qiu, Yuqing Jiao, Qiannan Wang, Limin Feng

**Affiliations:** 1 Department of Gynecology, Civil Aviation General Hospital, Beijing, China; 2 Department of Gynecology, The First Affiliated Hospital of Shandong First Medical University & Shandong Provincial Qianfoshan Hospital, Jinan, China; 3 Department of Gynecology, Beijing Chuiyangliu Hospital, Tsinghua University, Beijing, China; 4 Department of Gynecology, Beijing Tiantan Hospital, Capital Medical University, Beijing, China

**Keywords:** hysteroscopy, intrauterine adhesion, AFS scores, silicone rubber uterine, pregnancy outcome

## Abstract

**Background:**

Recurrent intrauterine adhesions (IUAs) after hysteroscopic adhesiolysis severely compromise fertility. This randomized trial investigated the efficacy of a pioneering silicone rubber uterine stent in preventing adhesion reformation.

**Methods:**

In a single-center, double-blind trial, 45 patients with moderate-to-severe IUAs underwent hysteroscopic adhesiolysis. The patients were randomized to receive a silicone rubber uterine stent (treatment group) or an auto-crosslinked hyaluronic acid gel. Intrauterine device (IUD) and 12-f Foley balloon catheter at the end of surgery (control group). All patients underwent second-look hysteroscopy at 2–3 months and hormonal therapy for two cycles after surgery. Pregnancies over 5 years were followed up.

**Results:**

Both groups had reduced American Fertility Society (AFS) scores postoperatively (*P* < 0.005), but the stent group achieved significantly greater reductions (*P* = 0.003). Severe IUA subgroup analyses revealed near-doubled AFS score improvements with stents versus controls (*P* = 0.023). The stent group demonstrated superior uterine cavity restoration, with markedly better involvement range scores (*P* < 0.05). The long-term data revealed that stent-treated patients had higher cumulative pregnancy rates (59.1% vs. 34.8%), with advantages emerging as early as 6 months post-surgery.

**Conclusion:**

Silicone uterine stents represent a breakthrough in preventing post-adhesiolysis IUAs, particularly for severe cases, offering enhanced anatomical recovery and fertility outcomes. This innovation addresses a critical unmet need in reproductive surgery, providing a robust strategy to safeguard fertility potential.

## 1 Background

Asherman syndrome (AS), first described by Asherman in 1950, is defined as partial or complete obstruction of the uterine cavity by adhesions secondary to trauma of the basal layer, resulting in menstrual abnormalities, cyclical lower abdominal pain, infertility, or recurrent pregnancy loss (1, 2). Intrauterine surgery and infection have been identified as the two main causes of this disease (3).

Hysteroscopy has become the standard method for diagnosing and treating intrauterine adhesions (IUAs) (4). The aim of transcervical resection of adhesions (TCRA) is to restore the normal uterine structure by removing the adherent tissue under direct vision. However, the readhesion rate after TCRA is as high as 20%–62.5% (5, 6). And the pregnancy rate is only 22.5%–33.3% in severely adherent patients, which seriously affects women’s reproductive health (7, 8). Therefore, compared with surgical removal of connected tissue, prevention of IUA reoccurrence after surgery is more challenging.

Medical silicone rubber, a non-toxic, non-irritating material with good biocompatibility, is gradually being used in the fields of breast implants and arthroplasty of the wrist (9, 10). The first exploration of the therapeutic efficacy of silicone rubber stents in the field of uterine adhesions was a case report in 2019 (11). A retrospective study demonstrated that silicone rubber stents are effective at preventing adhesion reformation after TCRA (12). In a recent study, the authors evaluated the pharmacokinetics and safety of silicone rubber stents as estrogen carriers in the rat uterus, providing a new idea for preventing IUA recurrence (13). However, there have been no clinical reports about the effects of silicone rubber stents on adhesion recurrence or pregnancy outcomes after TCRA.

**FIGURE 1:**
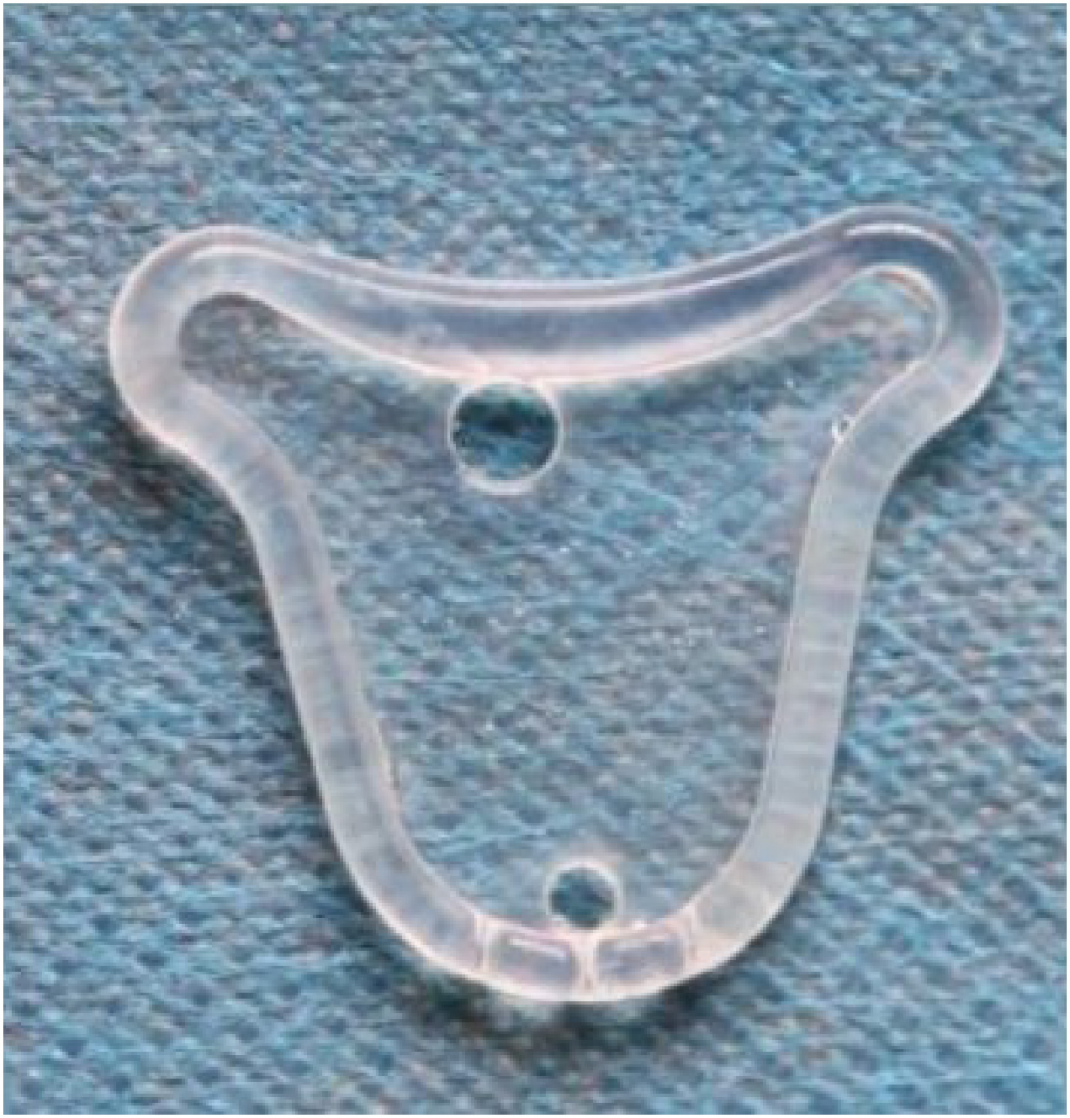
The silicone rubber uterine stent.

The silicone rubber stent is a uterine-type sheet device (Figure 1). The lower end of the stent is equipped with three drainage grooves, which is more conducive to the drainage of fluid in the uterine cavity and reduces the occurrence of intrauterine infection. In more than 30 years of prior clinical practice, autocross-linked hyaluronic acid (ACP) gel and Foley balloon catheters have been recommended in the European Society of Gastrointestinal Endoscopy practice guidelines as Grade A (14). It has also been reported that the combination of a Foley balloon catheter and an intrauterine device (IUD) may be more effective in preventing readhesion after TCRA than an IUD or Foley balloon catheter alone (15). However, the effects of these methods have been questioned. A previous study revealed that the release of copper ions produces an aseptic inflammatory response that may be detrimental to the treatment of uterine adhesions (16, 17). The Foley balloon catheter is inexpensive and simple to use, but the shape of the balloon does not match the uterine cavity, resulting in the inability to separate the adhesions on both horns of the uterus (18). Furthermore, prolonged intrauterine placement has raised concerns about the potential for increased rates of intrauterine infection.

We conducted a randomized controlled study to evaluate whether silicone rubber stents could prevent adhesion recurrence and improve pregnancy rates in patients with moderate to severe IUA.

## 2 Materials and methods

### 2.1 Study design

This clinical trial was registered on Chinese Clinical Trials Registry (URL: ClinicalTrials.gov) on 30 June 2021 (No: NCT05474756). In this prospective randomized controlled trial, participants were recruited between April 2019 and January 2021 from the Department of Gynecology of Tiantan Hospital, Beijing. The inclusion criteria were women aged 20–40 years with moderate to severe IUA [American Fertility Society (AFS) score ≥ 5], patients with a desire for future fertility and IUA as the only infertility factor. Patients were excluded if they had received estrogen therapy within 30 days before the procedure or had severe systemic disease or any contraindications to TCRA surgery.

The participants were randomly divided into two groups with a simple randomization sequence generated by a computer random number generator and maintained by a doctor who was not involved in patient registration. The sequence was not accessible to any researcher. Opaque sealed envelopes were used to store the documents and were opened by the gynecologist before the procedure. The AFS scores before treatment weas evaluated by the surgeon at the first hysteroscopy in all patients. The treatment group received a silicone rubber uterine stent (Haokang Medical Technology Co., Ltd., Hunan, China) at the end of surgery. The control group received 3 mL of ACP gel (MateRegen gel; BioRegen Biomedical Co., Ltd., Changzhou, China), IUD (YUANGONG COPPER IUD; YANTAI JISHENGYAOXIE CO., LTD, China) or a 12-f Foley catheter (UROCARE Sterile Urethral Catheter, URO TECHNOLOGY SDN. BHD) with a balloon filled with 2.5 ml of saline at the end of surgery. The doctor removed the urinary catheter on the first postoperative day. Both the silicone rubber uterine stent and the IUD were removed by grasping forceps under direct hysteroscopic vision at the time of the second hysteroscopy 2–3 months later. The second-look AFS score was determined following removal. All patients received standard care. Hormonal therapy consisting of 6 mg/day of estradiol valerate tablets (PROGYNOVA; Bayer) was started on the day of surgery for 25 days with the addition of progesterone (progesterone capsules; ZHEJIANG XIANJU PHARMACEUTICAL CO., LTD.) at an oral dose of 200 mg/day for the last 6 days of estrogen therapy.

### 2.2 Surgical procedure

All patients underwent TCRA during the proliferative phase of the endothelium, and surgical procedures were performed under general anesthesia. The procedure was performed by the same surgeon (M.D. Feng) via a surgical hysteroscope (8.5 mm, Olympus, Japan) and perfused with 0.9% saline at a flow rate of 300–340 ml/min at a pressure of 100–120 mmHg. Ultrasound guidance was routinely used. Once the extent and severity of uterine adhesions were assessed, bipolar instruments or scissors were used to separate the adhesions until normal uterine anatomy was restored.

### 2.3 Outcome measures

The primary outcome was the reduction in the AFS score before treatment, and the second outcome was the AFS score. The secondary outcome was the pregnancy rate within 5 years postoperatively, and follow-up was performed through outpatient or telephone consultation.

### 2.4 Sample size and power consideration

The primary endpoint of this study was the reduction in AFS score after surgery. Because no prior randomized data on silicone stents were available at the time of study design, a formal prospective sample size calculation could not be performed. Therefore, this trial was conducted as an exploratory randomized study with a target enrollment of approximately 20–25 participants per arm, which is consistent with previous IUA interventional studies of similar design. After completing data collection, a *post hoc* power analysis was performed based on the observed between-group difference in AFS score to evaluate the adequacy of statistical power for the primary outcome.

### 2.5 Statistical analysis

A Kolmogorov–Smirnov test was used to examine the data distribution. Numerical data with a normal distribution are presented as the means ± SDs, whereas data with a skewed distribution are presented as the medians (interquartile ranges). Variables with a normal distribution, such as age and baseline AFS score, were compared between groups using Student’s *t*-test, whereas skewed continuous variables, such as the reduction in AFS score in the severe adhesion subgroup, were compared using the Mann–Whitney *U*-test. The frequency distributions were compared via a chi-square test. A value of *P* < 0.05 was considered statistically significant. Cumulative pregnancy rates were compared using Kaplan–Meier survival analysis and the log-rank test. Cox proportional hazards models were used to estimate hazard ratios with 95% CI, we visualized the results via GraphPad Prism (version 10.1.2), which provided a clear representation. All the statistical analyses were performed via SPSS (version 25.0).

## 3 Results

### 3.1 Patient characteristics

**FIGURE 2:**
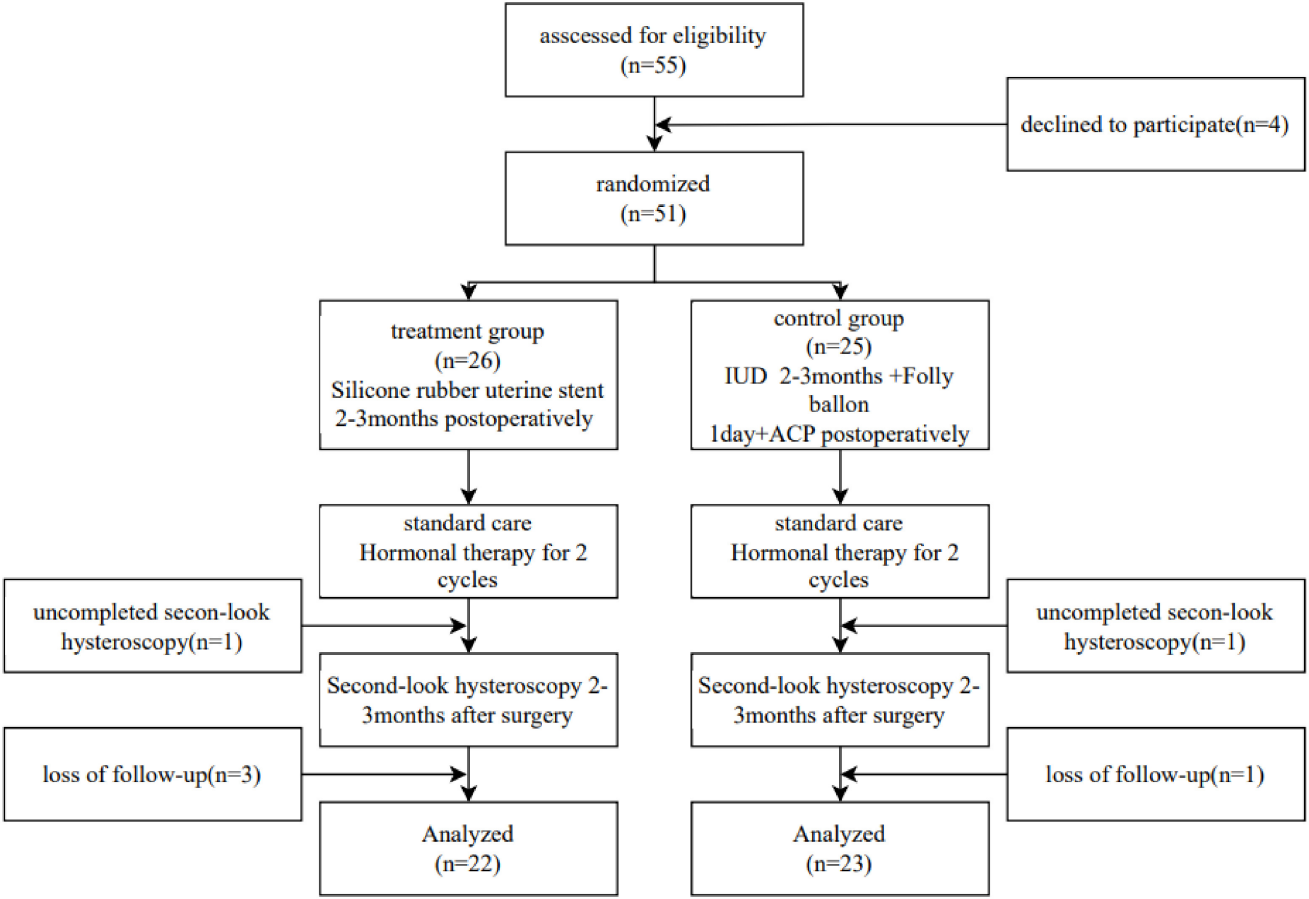
Flow diagram of the trial.

Among the 55 eligible patients, Figure 2 shows the flow chart of the study with annotated exclusions. Four declined participation before randomization, two withdrew during the study, and four were lost to follow-up, resulting in 45 patients who completed the study (22 in the treatment group and 23 in the control group) (Figure 2). The baseline characteristics of the patients who completed the study were similar across groups, and the characteristics of those who declined, withdrew, or were lost to follow-up were comparable to the analyzed population, suggesting that these losses are unlikely to have biased the primary or secondary outcomes, including pregnancy rates (Table 1).

**TABLE 1:** The clinical characteristics of each group.

Variable	Treatment group *N* = 23	Control group *N* = 22	*P*-value
Age (years)^a^	31.6 ± 3.6	31.6 ± 4.0	0.956
Body mass index^a^	22.8 ± 3.7	22.4 ± 4.2	0.755
Gravida^a^	1.8 ± 1.0	2.2 ± 1.5	0.255
Para^a^	0.2 ± 0.4	0.5 ± 0.5	0.106
Time interval (days)^a^	89.9 ± 18.0	97.4 ± 34.0	0.368
Time interval (day)^a^	90 ± 18	97 ± 34	0.363
AFS score^b^	8 (6–10)	8 (7–10)	0.934
History of uterine surgery^c^	–	–	0.132
Hysteroscopic surgery	2 (9.1%)	2 (8.7%)	–
D&C for induced abortion	16 (72.7%)	10 (43.5%)	–
D&C for induced abortion	4 (18.2%)	8 (34.8%)	–
Menstrual pattern^c^	–	–	0.673
Amenorrhea	3 (13.6%)	1 (4.3%)	–
Oligomenorrhea	17 (77.3%)	21 (91.3%)	–
Normal menses	2 (9.1%)	1 (4.3%)	–
Grades by AFS score^c^	–	–	–
Moderate	14 (63.6%)	16 (69.6%)	0.673
Severe	8 (36.4%)	7 (30.4%)	–

^a^Student’s *t*-test.

^b^Mann–Whitney *U*-test.

^c^Chi-square test and contingency table analysis. AFS, American Fertility Society; D&C, dilation and curettage. The values are presented as the means ± standard deviations, medians (interquartile ranges), or numbers (%), unless otherwise indicated. *P* < 0.05 was considered statistically significant.

None of the patients experienced perforation, infection, abnormal vaginal bleeding or other complications.

### 3.2 Comparison of AFS score reduction between the two groups

**FIGURE 3:**
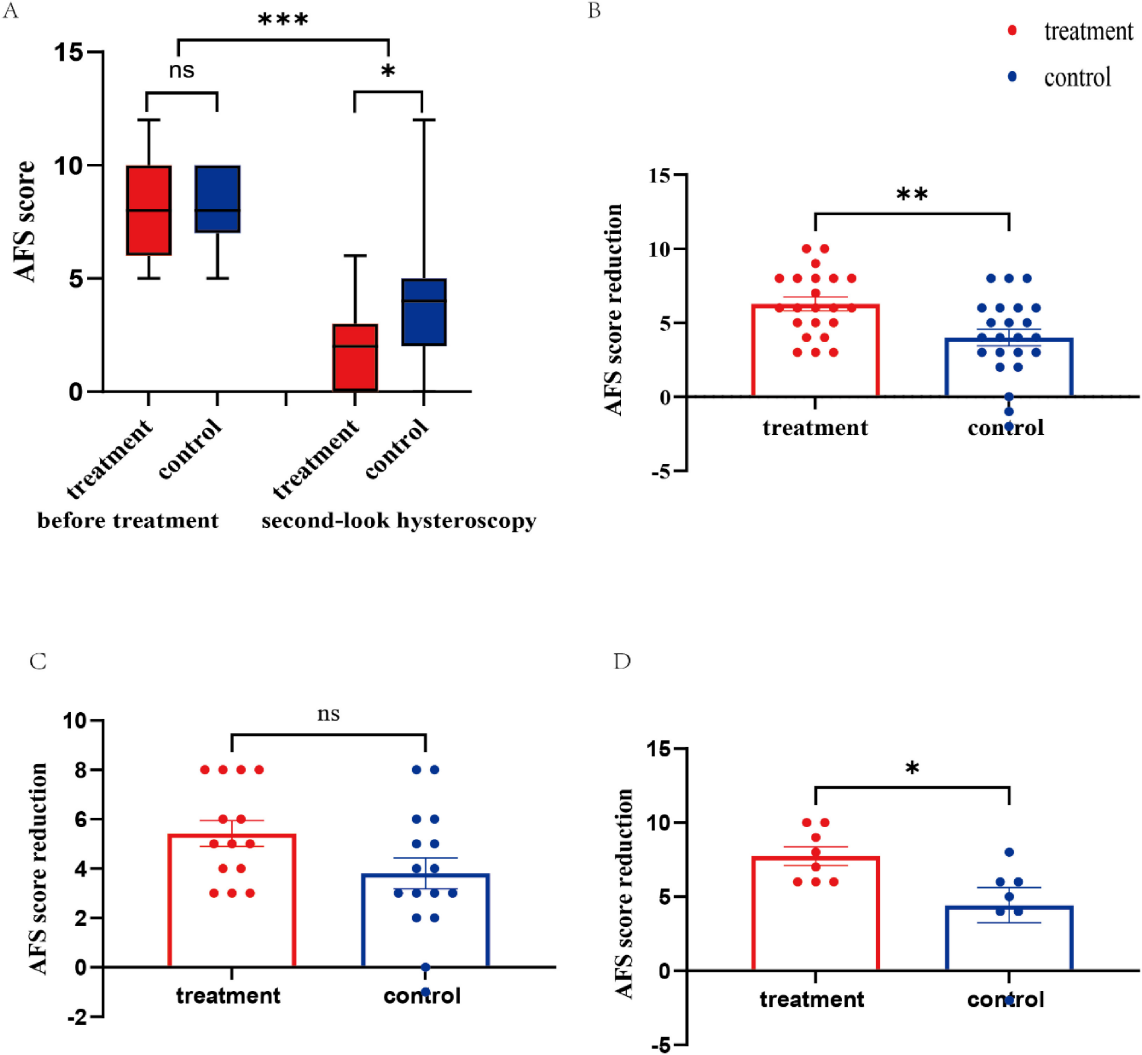
AFS score and the reduction in the scores before treatment and second-look AFS score. **(A)** Comparison of pre- and postoperative adhesion scores. **(B)** Reduction in the AFS score of the treatment group and control group. **(C)** Reduction in the AFS score of patients with moderate adhesion. **(D)** Reduction in the AFS score of patients with severe adhesion. AFS, American Fertility Society; NS, not significant. *P < 0.05, **P < 0.01, ***P < 0.001.

Compared with the AFS score before treatment, the second-look AFS score was significantly lower in both the treatment and control groups (Figure 3A). The AFS score at the second-look hysteroscopy in the treatment group was significantly lower than that in the control group (*P* = 0.003, Table 2). Furthermore, patients in the treatment group had greater AFS score reductions than did those in the control group (6.27 ± 2.16 vs. 4.00 ± 2.66, *p* = 0.003; Figure 3B). In patients with moderate adhesions, the AFS score was not significantly different between the two groups (5.43 ± 1.95 vs. 3.81 ± 2.50, *P* = 0.061; Figure 3C). However, in patients with severe adhesions, the reduction in AFS score was more significant in the treatment group than in the control group (7.75 ± 1.75 vs. 4.43 ± 3.16, *P* = 0.230; Figure 3D). According to the subgroup analysis of AFS scores, patients in the treatment group had greater extents of cavity involvement scores than did those in the control group in both the moderate and severe adhesion groups did (*P* < 0.05), whereas the types of adhesion scores and menstrual pattern scores were not significantly different (*P* > 0.05). A *post hoc* power analysis based on the AFS score reduction demonstrated adequate statistical power for the primary endpoint (power = 0.83 at α = 0.05), supporting the robustness of the main finding.

**TABLE 2:** Differences in adhesion grade between the two groups of women.

Outcome measures	Treatment group (*n* = 22)	Control group (*n* = 23)	*P*-values
Second-look AFS score	2 (0–3)	4 (2–5)	0.003
ΔAFS score			
Total^a^	6.27 ± 2.16	4.00 ± 2.66	0.003
Moderate adhesion^a^	5.43 ± 1.95[14]	3.81 ± 2.50[16]	0.061
Extent of cavity involved^b^	2 (1–2)	0 (0–2)	0.022
Type of adhesions^b^	2 (0.75–4)	2 (0–3)	0.615
Menstrual pattern^b^	2 (2–2)	2 (0–2)	0.225
Severe adhesion^a^	7.75 ± 1.75[8]	4.43 ± 3.16[7]	0.023
Extent of cavity involved^b^	3 (2.25–3)	0 (0–3)	0.049
Type of adhesions^b^	2.5 (1.25–3.75)	0 (0–3)	0.146
Menstrual pattern^b^	2 (2–3.5)	2 (0–2)	0.202

^a^Student’s *t*-test.

^b^Mann–Whitney *U*-test. The values are presented as the means ± standard deviations or medians (interquartile ranges). *P* < 0.05 was considered statistically significant.

### 3.3 Comparison of pregnancy rates among patients

**FIGURE 4:**
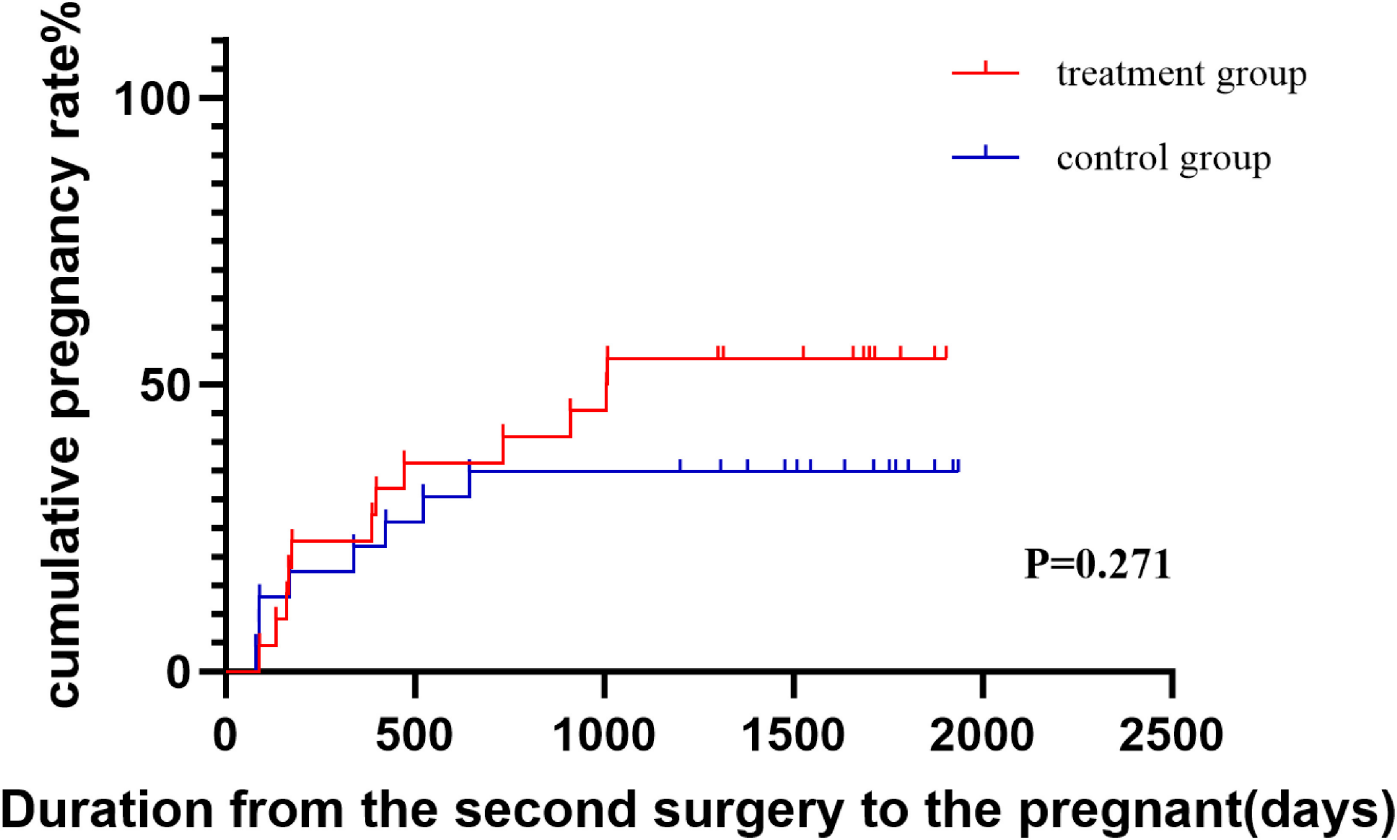
Cumulative pregnancy rate curves generated via the Kaplan–Meier method on the PR dataset.

The pregnancy rate in the treatment group was not significantly different from that in the control group (*P* = 0.182, Table 3). Cumulative pregnancy rates over the follow-up period were illustrated using Kaplan–Meier curves (Figure 4). The log-rank test showed a trend toward higher cumulative pregnancy probability in the treatment group compared with controls (*P* = 0.27). Cox regression analysis yielded an HR of 1.62 (95% CI: 0.71–3.72), suggesting a non-significant trend of increased likelihood of conception in the treatment group. These results indicate that the observed differences in pregnancy outcomes should be interpreted cautiously. Furthermore, the median gestation time from second surgery to conception was 33 months in the treatment group, and the control group was not followed up. According to the Kolmogorov–Smirnov curve, PR was greater in the treatment group than in the control group at any point after 6 months.

**TABLE 3:** Comparison of pregnancy rates between the treatment group and the control group.

Group	Pregnant	No pregnant	Total	*P*-value
Treatment group	12 (54.5%)	10 (45.5%)	22	–
Control group	8 (34.8%)	15 (65.2%)	23	182^a^
Total	20	25	45	–

^a^Chi-square test and contingency table analysis. The values are given as numbers (%). *P* < 0.05 was considered statistically significant.

## 4 Discussion

This is the first prospective randomized controlled study to explore whether intrauterine placement of silicone rubber stents after TCRA affects IUA reformation and clinical pregnancy rates. The treatment group had the advantage of reducing the extent of cavity involvement scores, and patients with severe adhesions were more likely to benefit from the use of silicone rubber stents after TCRA.

Takasaki K first retrospectively demonstrated that the use of silicone rubber stents was effective in preventing adhesion reformation after TCRA, which is consistent with our conclusions (19). The silicone rubber stent used in this study has the advantages of conforming to the morphology of the uterine cavity, does not compress endometrial blood flow, and the caudal groove facilitates the drainage of uterine fluid.

The use of silicone rubber stents significantly reduced the extent of cavity involvement scores. This may be because the silicone rubber stent conforms to the morphology of the uterine cavity. The IUD is limited to only the periphery of the cavity of the uterus. Although the combined use of a Foley balloon prevents contact between the anterior and posterior walls of the central uterus, it was designed to be retained only 1 day after TCRA, in view of the discomfort and the risk of uterine infection caused by the tail of the catheter being left outside of the vagina. Related studies have reported that ACPs can be retained in the uterine cavity for at least 3 days (20). However, it has been reported that 2 months are needed for complete endometrial wound healing following hysteroscopic surgery (21). This finding suggested that the duration required for the barrier to prevent adhesion reformation after TCRA was at least 2 months. Thus, the control method is clearly insufficient to achieve continuous separation of the anterior and posterior uterine walls. In contrast, silicone rubber stents separate the anterior and posterior walls of the peripheral and central uterine cavities and can be retained for 2–3 months after TCRA.

The pregnancy rate in the treatment group was 54.5%, which was higher than the 33.3% previously reported in the literature. Fibrous tissue within the uterine cavity affects sperm entry into the fallopian tubes. The decreased receptivity of the endometrium impacts the implantation of the zygote (22). The silicone rubber stent prevents direct contact between the anterior and posterior walls of the uterus, and when combined with periodic estrogen treatment, it promotes scarless repair and re-epithelialization (23, 24). As a result, the pregnancy rate in the treatment group was higher than that reported in previous studies. The treatment group had better pregnancy potential after 6 months. Adhesions recur over time, and the extent of adhesions in the treatment group was less than that in the control group. We speculate that the difference in uterine cavity volume affects the pregnancy rate after 6 months. Although the study was sufficiently powered for the primary outcome, the sample size was not based on pregnancy outcomes. The treatment group showed a trend toward higher cumulative pregnancy rates, but the difference did not reach statistical significance. The Kaplan–Meier and Cox analyses suggest a potential benefit, but given the limited sample size and exploratory nature of this secondary outcome, therefore, fertility results should be interpreted as exploratory. In addition, we note that the small sample size limits the ability to detect rare adverse events, and larger studies would be required to fully assess the safety profile of long-term stent placement.

## 5 Conclusion

In summary, the use of silicone rubber uterine stents can effectively prevent adhesions after TCRA. Patients with severe adhesions are particularly more likely to benefit from the use of silicone rubber stents after TURA. Although the pregnancy rate in the treatment group did not significantly improve compared with that in the control group, the pregnancy potential in the treatment group 6 months after surgery was significantly better.

## Data Availability

The original contributions presented in this study are included in this article/supplementary material, further inquiries can be directed to the corresponding author.
